# Tocilizumab Was Effective in Repairing the Large Geode in a Patient with Rheumatoid Arthritis

**DOI:** 10.1155/2020/8899391

**Published:** 2020-08-24

**Authors:** Takuya Izumiyama, Yu Mori, Eiji Itoi

**Affiliations:** Department of Orthopaedic Surgery, Tohoku University Graduate School of Medicine, 1-1 Seiryo-machi, Aoba-ku, Sendai, Miyagi 980-8574, Japan

## Abstract

Rheumatoid arthritis is characterized by multiple chronic arthritis subsequently inducing joint destruction. Although subchondral geode is a well-known feature of high-disease activity, a large geode is rare. Moreover, the treatment effect of biologic agents in the repair of large geode has not been reported. The present report shows the significant effect of interleukin-6 receptor blocker, tocilizumab, in repairing the large geode in the left humeral lateral epicondyle. This case implies that tocilizumab might be an effective treatment in patients with rheumatoid arthritis even with large geode.

## 1. Introduction

Rheumatoid arthritis (RA) is characterized by multiple chronic arthritis leading to bone erosion, joint malalignment, bony fusion, and joint destruction [1]. To prevent joint damage and decline in daily life activity, we should treat RA tightly as soon as possible because joint destruction is likely to progress in the early stage of the disease [2]. However, some patients present with bony erosion and joint destruction because of poor symptom management and/or poor prediction of severity [3]. Although subchondral geode is a well-known feature especially in high-disease activity, large geode is rare [3]. In previous studies, the large geodes mainly developed in the proximal tibia and femur [4–7], and most patients with high risk of fracture mostly underwent surgical treatment [4, 8–10]. Moreover, the treatment effect of biologic agents in the repair of large geode has been not reported. In this report, we present the significant effect of interleukin- (IL-) 6 receptor blocker, tocilizumab, in repairing the large geode in the left humeral lateral epicondyle.

## 2. Case Report

**Figure 1:**
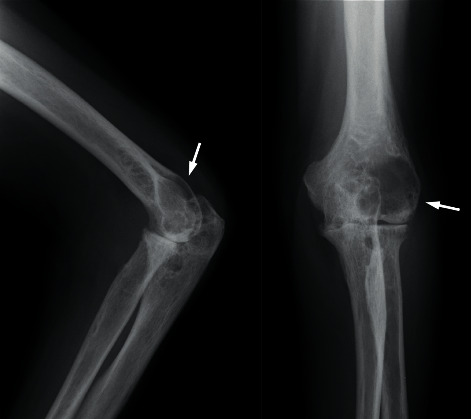
Plain radiograph of the left elbow obtained at the first visit to our department. A large transparent area is observed in the posterior lateral humeral bone (arrow). The vertical range is 29.8 mm, and lateral range is 33.0 mm in the anteroposterior view.

**Figure 2:**
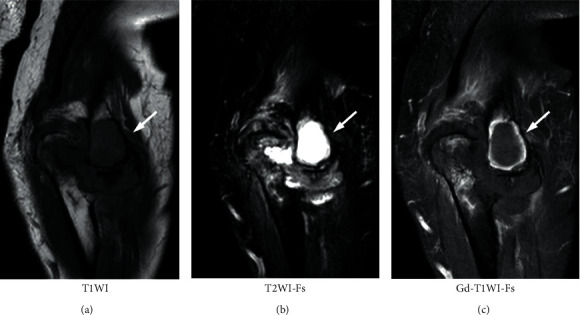
Magnetic resonance imaging of the left elbow obtained at the first visit to our department. (a) T1-weighted image (T1WI); (b) fat-suppressed T2-weighted image (T2WI-Fs); and (c) gadolinium-enhanced and fat-suppressed T1-weighted image (Gd-T1WI-Fs). A large low-intensity area is observed in T1WI, and high-intensity area is observed in T2WI-Fs. In Gd-T1WI-Fs, a large low-intensity area enhanced peripherally is noted (arrow). It seems as a large cyst surrounded by synovium.

**Figure 3:**
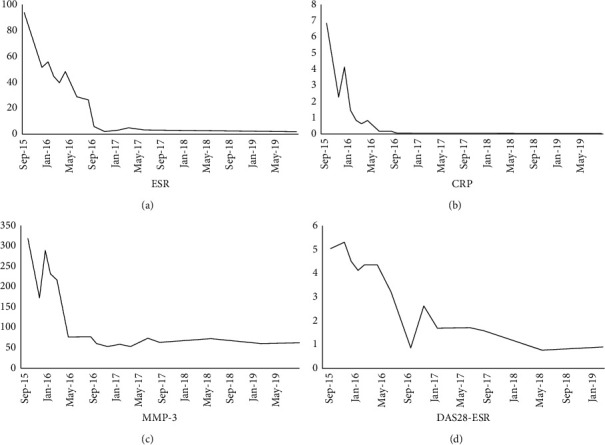
Transition of (a) erythrocyte sedimentation rate (ESR), (b) C-reactive protein (CRP), (c) matrix metalloproteinase (MMP)-3, and (d) disease activity score (DAS) 28. After visiting our department, the patient was treated with methotrexate. From August 22, 2016, the patient started to receive tocilizumab, and all laboratory data and DAS28 improved remarkably and immediately after administration.

**Figure 4:**
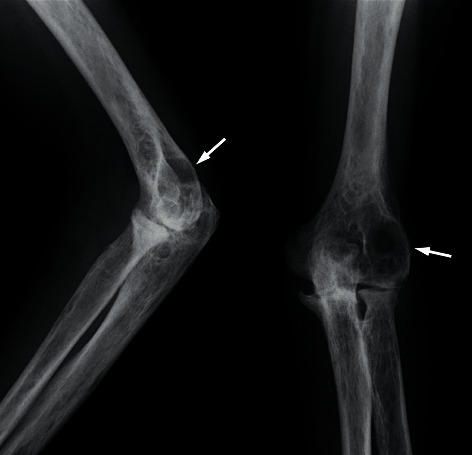
Plain radiograph of the left elbow at 10 months since the start of oral methotrexate. The large transparent area still remained, and poor repair is noted in the posterior lateral humeral bone (arrow).

**Figure 5:**
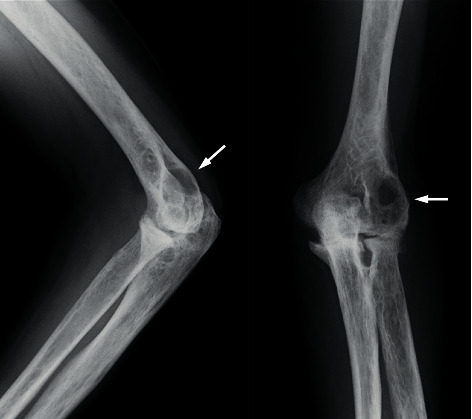
Plain radiograph of the left elbow at 10 months since the administration of subcutaneous tocilizumab injection. The transparent area gradually decreased, and bone formation progressed from the peripheral area (arrow). The size of evident geode is 10.8 mm × 8.3 mm.

**Figure 6:**
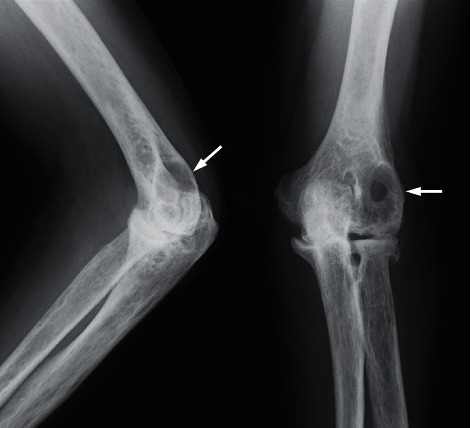
Plain radiograph of the left elbow at one month after administration of denosumab every three months. The transparent area showed little improvement effect compared to Figure 5 (arrow).

**Figure 7:**
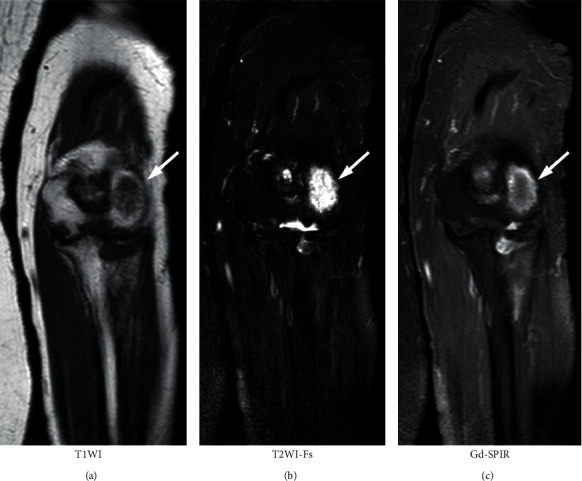
Magnetic resonance image of the left elbow, 3 years 5 months after the first visit to our department. (a) T1-weighted image (T1WI); (b) fat-suppressed T2-weighted image (T2WI-Fs); and (c) gadolinium-enhanced short T1 inversion recovery image (Gd-SPIR). The high-intensity area appeared from the peripheral cavity and internal low-intensity area became slightly brighter in T1WI. The peripheral high-intensity area was considered to demonstrate replaced bone marrow fat. The high-intensity area is noted in T2WI-Fs, but the size and signal strength decreased. In Gd-SPIR, the low-intensity area became slightly brighter, and the peripherally enhanced area seemed to be weaker (arrow).

A 66-year-old Japanese male patient with bilateral elbow pain and swelling was suspected of having bone tumor in the left humeral lateral epicondyle by radiography in an orthopedic clinic and referred to our department. However, bone tumor was denied by experienced bone and soft tissue tumor surgeons with magnetic resonance imaging (MRI). The subjective symptoms were swelling and pain of the bilateral elbow and left wrist. The left elbow had deformity with neither redness nor local heat. The laboratory examination showed erythrocyte sedimentation rate of 94 mm, C-reactive protein level of 7.0 mg/dL, rheumatoid factor of 70.2 IU/dL, and anticyclic citrullinated peptide antibody level of 156.8 U/mL. Plain radiography showed a 29.7 mm × 21.0 mm of large geode area in the left humeral lateral epicondyle (Figure 1). MRI demonstrated a large lesion, which had high intensity in T2-weighted images, low intensity in T1-weighted images, and enhanced peripheral area of the geode in gadolinium-enhanced T1-weighted images (Figure 2). These images suggested that the cyst consisted of fluid with peripheral synovitis. The patient fulfilled 2010 ACR/EULAR classification criteria for RA [11] and was diagnosed with RA. Methotrexate was administered, and the dose was gradually increased to 12 mg/week. However, the disease activity score (DAS) 28 was still high (Figure 3), and radiography indicated no evident improvement of the large geode in the left humeral lateral epicondyle (Figure 4). Therefore, 162 mg of tocilizumab was administered every 2 weeks. After subcutaneous injection of tocilizumab, the patient reported articular pain, laboratory data showed inflammation factor, and DAS28 immediately improved (Figure 3). Furthermore, the large geode in the left humeral lateral epicondyle gradually improved, and progression of bone formation from peripheral area was shown in radiography (Figure 5). The area of geode was significantly decreased to 10.8 mm × 8.3 mm. As additional treatment, denosumab was administered by subcutaneous injection every three months. However, additional denosumab showed no eminent effect on geode repair (Figure 6). In contrast, MRI showed decreased area in the geode and diminished peripheral contrast enhancement effect compared to MRI before tocilizumab administration (Figure 7). Left elbow pain significantly ameliorated, and activity of daily life improved without surgical treatment.

## 3. Discussion

This case showed eminent repair of the large geode by tocilizumab in a patient with RA. Several reports revealed that biological disease-modifying antirheumatic drugs (DMARDs) could repair the bone erosion [12–15]. However, to the best of our knowledge, the treatment effect on the large geode has not been reported. Although several previous studies described the large geode in RA [9, 10, 16], surgeries were performed immediately after diagnosis because of existing fracture or preventing the risk of fracture [9, 10, 16, 17]. Tocilizumab administration may be an effective treatment for large geode and one of the useful treatment alternatives before performing surgeries.

Our strong interest is whether tocilizumab has an additional treatment effect other than anti-inflammatory effect. The positive effect for bone metabolism related with anti-IL-6 therapy is still unclear; nevertheless, some studies showed that tocilizumab increases bone formation marker and decreases bone resorption marker [18–22]. Kume et al. showed the improvement effect on bone mineral density (BMD) in patients with RA treated with tocilizumab [23]. Furthermore, Chen et al. showed the decrease of the bone resorption marker and BMD improvement with tocilizumab treatment [24]. These anti-bone resorption effects of tocilizumab may explain the repairing effect of the large geode. Denosumab indicated the effect of the bone erosion repair by strongly inhibiting osteoclast activity [25]; therefore, we added denosumab for the acceleration of bone repair. However, expected effects were not obtained in this case. These results suggested that suppressing inflammatory synovitis might be more effective in geode repair compared to inhibiting osteoclast activity in patients with RA with large geode.

Another question is whether other biological DMARDs, including anti-tumor necrosis factor- (TNF-) *α* and anti-CD80/86, have the same improvement effect on the large geode. Several studies revealed that anti-TNF-*α* improves bone metabolism and BMD [26, 27]. A few reports showed the positive effect of anti-CD80/86 treatment on bone metabolism [28, 29]. Biological DMARDs potentially have the positive effect on bone effect repair. However, to our best knowledge, similar studies have not been published.

Therefore, we showed the eminent effect of tocilizumab on the large geode repair. Considering tocilizumab as an alternative treatment for patients with large geode may be helpful in the prevention of fracture, and consequently, patients with RA may have the chance of maintaining activities of daily living.

## Data Availability

All data generated or analyzed during this study are included in this published article.
